# Shiftwork-Mediated Disruptions of Circadian Rhythms and Sleep Homeostasis Cause Serious Health Problems

**DOI:** 10.1155/2018/8576890

**Published:** 2018-01-21

**Authors:** Suliman Khan, Pengfei Duan, Lunguang Yao, Hongwei Hou

**Affiliations:** ^1^The Key Laboratory of Aquatic Biodiversity and Conservation of Chinese Academy of Sciences, Institute of Hydrobiology, Chinese Academy of Sciences, Wuhan, Hubei 430072, China; ^2^Collaborative Innovation Center of Water and Security for Water Source Region of Mid-Line of South-to-North Diversion Project, College of Agricultural Engineering, Nanyang Normal University, Nanyang, Henan, China

## Abstract

Shiftwork became common during the last few decades with the growing demands of human life. Despite the social inactivity and irregularity in habits, working in continuous irregular shifts causes serious health issues including sleep disorders, psychiatric disorders, cancer, and metabolic disorders. These health problems arise due to the disruption in circadian clock system, which is associated with alterations in genetic expressions. Alteration in clock controlling genes further affects genes linked with disorders including major depression disorder, bipolar disorder, phase delay and phase advance sleep syndromes, breast cancer, and colon cancer. A diverse research work is needed focusing on broad spectrum changes caused by jet lag in brain and neuronal system. This review is an attempt to motivate the researchers to conduct advanced studies in this area to identify the risk factors and mechanisms. Its goal is extended to make the shift workers aware about the risks associated with shiftwork.

## 1. Introduction

Fast growing needs demand doing work in recurring periods other than the traditional diurnal periods. The rotations in working shifts disrupt natural sleep-wake cycle and eating patterns [1], which in turn cause serious health problems by affecting mental health and work effectiveness [2]. This disruption alters circadian rhythms [3] and neuronal functions to cause neuronal disorders [2]. Shiftwork for a long period increases the risk of fatigue, aggression, sleep disorders, metabolic disorders, mental abnormalities, and death [2, 4–9]. Shiftwork directly affects alertness [10, 11], causes depression, and promotes anxiety [12].

Working repeatedly during night shifts affects hormonal system and disrupts hormonal secretions and their control factors [13–16]. This altered hormonal profile increases the risk of breast cancer, prostate cancer, gastrointestinal abnormalities, cardiovascular diseases, and reproductive aberrations [17–19]. Alterations in physiological, behavioral, and psychological mechanisms [5] further develop abnormalities associated with peptic ulcer, diabetes type II, and rheumatoid arthritis [20–22]. The measurement of shiftwork is considered complex as it requires several parameters including sleep quality, fatigue level, types of sleep problems, use of stimulants, and sleepiness [2, 23].

Shiftwork has gained huge importance which is inevitable in modern world. This situation alerts researchers to focus on shiftwork and related abnormalities. Without knowing the genetic mechanisms and identifying factors related to shiftwork-promoted health problems, development of prevention and curing strategies may not be achievable. Suitable model organism, monitoring systems, and mimicking the shiftwork in lab conditions are major challenges in studying shiftwork to investigate related disorders. These challenges make the identification genes, majorly involved in shiftwork-related abnormalities, difficult. In this review, we have focused on major health problems that are linked with shiftwork directly or indirectly.

## 2. Shiftwork Dysregulates Circadian Rhythms by Affecting Clock Genes

Circadian rhythm mainly controls the daily wake and sleep cycle and regulates physiological processes including hormone secretion, body temperature, feeding behavior, cell cycle progression, and drug, glucose, and xenobiotic metabolism. Its disruption through environmental and genetic means causes aberrations in physiological processes. Clock genes with the effects of oscillators and endocrine and neural signals regulate circadian rhythm [24] which may be disrupted apparently by shiftwork. Circadian clock, through appropriate physiological activities in relevance to time, controls the circadian rhythms [4]. Shiftwork (chronic jet lag) suppresses the expression levels of core clock genes, including Per1 and Per2 in suprachiasmatic nuclei (SCN) and MT1 melatonin and glucocorticoid receptors in the liver. It further causes delay in acrophases of circadian expression of Per1, Per2, BMAL1, and Dbp in liver. Besides clock genes, expression levels of some cell cycle-related genes including c-Myc and p53 are also altered [25]. Vasopressin (V1a and V1b) receptors (expressed in SCN neurons) promote shiftwork effect in combination with core clock genes. Individuals lacking these receptors are normally resistant to jet lag/shiftwork effects [26].

Circadian oscillator period is determined around 24 hours genetically and adjusted by synchronizers such as LD cycle. Circadian rhythms synchronized to day time work and night time sleep require phase adjustment in altered routines by central and peripheral oscillators. This phase adjustment in certain cases disrupts the normal organization and sequence of the clock. Clock genes behave differently during phase shifts [1, 27]. Shift workers undergo circadian dysrhythmia that adversely effects mental health. This further leads to circadian rhythm disorder causing aberrations in neurogenesis and spatial cognition [28].

Many aspects of circadian rhythmicity can be modulated by serotonergic agents that indicate that serotonin is involved in the regulation of circadian rhythm. Serotonin transporter (5-HTT) control serotonin reuptake depending on serotonin transporter gene (*SLC6A4*) promoter region (5-HTTLPR) [29]. Significant associations between shiftwork and S variant of the SLC6A4 promoter and 5-HT and 5-HIAA contents of platelet can help in investigating the circadian rhythm-related mechanisms imposed by shiftwork [29]. The effect of shiftwork on circadian rhythm and sleep may cause the metabolic dysregulation and depressions by affecting the genetic pathways. The affect may lead to the disruption of other functioning systems and cause relative disorders either through direct or indirect genetic interaction in continuous/discontinuous forms.

## 3. Shiftwork Disrupts Normal Sleep and Behavior

Sleep consists of two repeated cyclic patterns “nonrapid eye movement and rapid eye movement” [30]. It is controlled genetically with the influence of environmental factors [31, 32]. Adenosine, a sleep-promoting molecule [33], mediates wake-promoting effects of caffeine by acting on adenosine receptors antagonistically [34]. GABA (gamma-aminobutyric acid) promotes sleep whereas dopamine, acetylcholine, norepinephrine, and histamine promote wakefulness [35]. The cyclic guanosine monophosphate (cGMP) kinase [36, 37], regulatory subunit of Shaker [38], Sleepless (sss) gene [39], and CLK and CYC proteins [40] are key players in sleep regulation. All these genes and regulators may get disrupted in shift workers which will lead to abnormal sleep and other serious health conditions.

**Figure 1:**
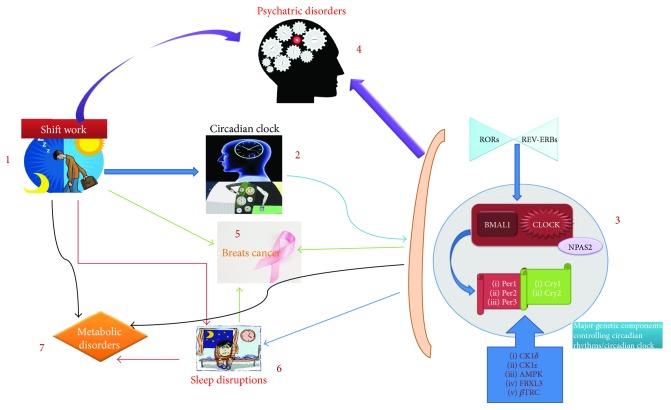
The abnormalities associated with shiftwork. (1) Shiftwork primarily affects the circadian clock and leads to several disruptions and disorders. (2) Disruption in circadian clock further affects the circadian system and alters important gene expressions which play an important role in maintaining normal body functions. (3) BMAL1 and CLOCK genes are the key factors that control circadian rhythms. Any alteration in these groups of genes further alters the genetic expression of genes (Per 1–3 and Cry 1/2) involved in clock maintenance. Other factors and proteins which play a major role in circadian rhythm are RORs, REV-ERBs, CK1*δ*, CK1*ε*, AMPK, FBXL3 and *β*TRC. (4) Shiftwork either directly or through circadian system alterations may cause severe psychiatric disorders including major depression, anxiety, and mood disorders. (5) Due to exposure to light irregularly, shiftwork enhances the chances of breast cancer. Breast cancer development is promoted by sleep disruptions, circadian system imbalances, and dietary conditions. (6) Sleep disorders and disrupted sleep is another condition developed by shiftwork. (7) This disruption in sleep affects the metabolism and causes metabolic disorders including obesity.

Shiftwork due to the rotation of working schedules and light/dark disturbance directly affects sleep (as shown in Figure 1) and may cause health abnormalities including insomnia, sleep apnea, periodic leg movements, restless leg syndrome (RLS), and sleep-wake state dissociation disorders such as rapid eye movement (REM) and narcolepsy, sleep behavior disorder, and sleep walking [34]. Furthermore, the shiftwork-induced deficits in sleep homeostasis and circadian rhythms may lead to different psychiatric disorders and affect SUR2 gene, which was found involved in energy metabolism and aetiology of cardiomyopathies [41]. Shiftwork may disrupt daily patterns of human physiology controlled by circadian rhythms and sleep, including regulation of energy patterns expenditure [42, 43] and glucose metabolism [42].

Shiftwork may negatively affect genes and factors involved in sleep disorders such that a SNP marker [44] and chemokine (C-C motif) receptor 3 (*CCR3*) susceptibility gene [45] associated with narcolepsy, MEIS1 locus, and neuronal nitric oxide synthase (NOS1) and BTBD9 associated with RLS [46–48]. Serine to glycine mutation in *PER2* and mutation in casein kinase I*δ* gene (*CSNK1D)* develop familial advanced sleep phase syndrome (FASPS) [34, 35] whereas point mutation in *PRNP* causes *fatal familial insomnia (FFI)* [35]. The disrupted functions of the above genes through shiftwork may lead to related disorders. Shiftwork can alter the functionality of sleep-promoting immune genes [49], glucocorticoids and NSAIDs [50], protein NF-kB (upregulated during sleep deprivation) [34], TAK1 (TGF-b-activated kinase) and *Sik3* (control sleep behaviors), and *Nalcn* gene (involved in REMS) [51]. Widening its effectiveness, shiftwork promotes acute coronary syndrome [52], menstrual disturbances, and abnormal insulin levels [53] and increases sleepiness and alcohol consumption [54]. It also lifts sleep deprivation that ultimately leads to loss in cognitive, physical, and metabolic consequences. These alterations can possibly end up with developing cardiovascular morbidity and mortality [29].

## 4. Shiftwork Dysregulates Metabolic Process and Develops Related Disorders

Social jet lag is a key player in developing metabolic syndrome by inducing changes in cholesterol levels and disrupting normal food processes. It was observed in an experiment that social jet lag potentiated body weight gain by increasing overconsumption of cafeteria food. As a result, it promoted high insulin and dyslipidemia indicating the risk of metabolic syndrome [55]. A chronic shift in light/dark (LD) cycles induces obesity, increases body weight and glucose intolerance, and accumulates more fat in white adipose tissues. It changes the expression profiles of metabolic genes in liver [56].

According to timed sleep restriction (TSR) study, sleep timing directly affects clock-controlled genes including ClockD19, BMAL1, Per1, Rev-erb*α* and Dbp, and circadian machinery. This alteration affects metabolic processes including carbohydrate and lipid utilization in liver. BMAL1, Per1, and Dbp were upregulated during early light phase and Rev-erb*α* during mid-light phase. Carbohydrate regulators affected were insulin receptor substrate 2 (Irs2), glucose-responsive fork head box O1 (Foxo1), and glucose transporter 2 (Slc2a2). These regulators control the functions of pyruvate carboxylase, the pyruvate transporter Slc16a7, pyruvate dehydrogenase kinase 4, the glycerol transporter aquaporin, fructose-2,6-biphosphatase 1, and liver pyruvate kinase. These alterations in carbohydrate regulators further affect glycerol kinase and glycerol phosphate dehydrogenase 2, glutamic-pyruvate transaminase, and regulators of glycerol biosynthesis [4]. This dysregulation may induce critical alterations in genetic system to develop metabolic disorders.

The presence of metabolic process controlling genes in rhythmic transcriptome indicates that alteration in circadian rhythm causes disruption in metabolic process [4, 57–59]. CLOCK, BMAL, and PER2 were found associated with obesity by affecting the metabolic process [60, 61]. Shiftwork brings about changes in appetitive hormones through circadian misalignment which causes reduction in leptin level that ultimately leads to weight gain. Shift workers are considered at higher risk of type II diabetes [42, 62].

## 5. Shiftwork-Related Health Risks

### 5.1. Shiftwork Is Associated with Mortality

Quick return occurs due to rotations in between shifts, causing major accidents [22] that normally lead to death. Shiftwork increases the risk of several disorders including mental disorders and sleep-related disorders, which may lead to death. In an experiment related to jet lag effects on transgenic aged rats, it was found that mortality light scheduled rotations at 6 hours phase advance, each week. The survival rate of rats exposed to shifted light schedule was found lesser (47%) than the survival rate (83%) of rats exposed to normal light (12L/12D) scheduled rotations. Although jet lag has no mortal effect on younger mice, it alters the behavior and circadian rhythms which further causes serious health issues by affecting brain and liver [7].

### 5.2. Shiftwork Induces Mental and Related Disorders

The role of SLC6A4 (serotonin transporter gene) in shift workers was confirmed to be related with time period. The proportion of short allele becomes higher than large allele if the period is more than 5 years. The effectiveness of shiftwork becomes higher in case of internal desynchronization of circadian rhythm [2, 63].

Endocrine imbalance in depression and psychosis is the hyperactivity of the HPA axis [64]. Glucocorticoid functions as an immunosuppressant and interacts with melatonin in a form of suppressive effect on the production of ACTH-induced glucocorticoid [65]. Its resistance results in hypercortisolemia in psychiatric patients and increased pituitary volume in depressed patients [66, 67]. Shiftwork causes desynchronization in circadian rhythm which in turn leads to reduced NK activity and weakening cellular immunity [68], disruptions in norepinephrine, melatonin, and serotonin production [69, 70] which may induce anxiety, depression, hypercortisolemia, and psychosis.

### 5.3. Shiftwork Disorder and Associated Factors

Shiftwork disorder is characterized by insomnia and excessive sleepiness which develops due to working schedules overlapping sleeping time [71]. According to a study, shiftwork disorder was found to occur frequently in males as compared to females, working during the night. Approximately, 9% of the night shift workers reported severe shiftwork disorder while one-third of the total had mild symptoms [71]. Shiftwork sleep disorder changes the behavior and sleeping periods permanently which could possibly be recovered through advanced therapies only. Treatment with modafinil, an effective compound against narcolepsy and obstructive sleep apnea, was found ineffective [6], suggesting that the mechanisms involved in shiftwork sleep disorder are not related to sleep apnea and narcolepsy. Molecular level studies with a broad range of patients, to investigate mechanisms, are needed to find out the main alterations in the system to prevent and cure the conditions properly.

The high number of nights and age were found associated with shiftwork disorder [22], whereas genotypes were found to be associated with morningness and eveningness. Allele 3111C is associated with extreme eveningness and shiftwork with semidominant influence on sleep phases without having an obvious influence on morning/evening preferences. 3111C/C homozygotes are associated with delayed shift of sleep [72] where melatonin can phase-shift the circadian clock being chronobiotic and a sleep promoting agent [73].

### 5.4. Shiftwork Sleep Disorder

Shiftwork sleep disorder is a condition defined by excessive sleepiness or insomnia accompanied by total sleep time reduction. It is 10–38% prevalent in shift workers [5, 43]. The disturbance in general is the sleep-wake cycle distortion of extrinsic origin. This disorder is related to the night and early morning timings. Reduction in alertness and performance along with the linkage to higher rates of comorbidity with GI disorders [67] make SWSD a severe and attention-requiring condition. Sleepiness during the night and insomnia during the day become more severe with continuous shiftwork for longer periods. Depression, ulcers, and sleepiness-related accidents are the ultimate risks associated with shiftwork sleep disorder. This sleepiness behavior during night is similar to the day time sleepiness in people with narcolepsy [6].

### 5.5. Advanced and Delayed Sleep Phase Syndrome

Advanced sleep phase syndrome (ASPS) is characterized by 3-4 hours advanced awakening times and sleep onsets relative to normal times. It is inherited in autosomal dominant mode, caused by circadian cycle irregularity as circadian clock genes are key players in its development. Missense mutations S662G (occurs in phosphorylation site within CSNK1-binding domain of PER2) and CSNK1D are involved in ASPS [58].

Delayed sleep phase syndrome (DSPS) is characterized by chronic inability to fall asleep and awaken at normal timings [58]. Circadian system genes are majorly involved in this dysregulated sleep behavior. Significant associations with T3111C polymorphism in the 3′UTR of CLOCK and the association of a SNP in the 5′UTR of PER2 with morning preference have been reported in DSPS [72, 74]. According to a study, amino-acid substitution S408N in the CSKN1E gene might protect the body form DSPS and non-24-hour sleep-wake syndrome development [58]. Both the shorter and longer VNTR (variable number tandem repeat) alleles were found (PER3-4/4) associated with DSPS [75]. Per3 gene is involved in delayed sleep phase syndrome and extreme diurnal preference.

Mutation in hPER2 has a notable association with advanced sleep phase syndrome, whereas some haplotypes of hPER3 have shown an association with delayed sleep phase syndrome. These conditions arise because of the alterations into casein kinase I*ε* (CKI*ε*) phosphorylation of the target clock proteins, affecting morningness and eveningness. Polymorphisms in 3′ flanking region of the human clock homolog (3111T/C, hClock) is associated with eveningness and morningness such that 3111T allele has lower evening preference than 3111C allele [72]. As shiftwork has direct impacts on sleep, it may affect the normal functioning of genes to cause one of the two sleep abnormalities ASPS and DSPS.

### 5.6. Circadian Rhythm Sleep Disorder

Circadian rhythm sleep disorder refers to the abnormalities brought about by circadian misalignment due to variations in sleep-wake pattern. The shift in sleep-wake time is commonly caused by shiftwork, jet lag, light exposure, and insufficient sleep period. Circadian misalignment alters neuroendocrine physiology, impairs glucose tolerance, and reduces insulin sensitivity [42, 62, 76, 77]. Sleep deprivation problem rises with the incompatibility between circadian rhythms and working periods [78]. Both circadian alterations and sleep deprivations lead to fatigue, impairments in vigilance and attention, sleepiness [79], sleep deficiency, impaired physiological function, and aberrant behavior [76, 80–82]. A subset of insomnias including non-24-hour sleep-wake syndrome is also linked with circadian rhythm sleep disorder [81]. Exposure to bright light in shiftwork and working in consecutive shifts if maintained for longer time may change the system at the genetic level. This alteration will further disrupt the normal functions of gene-related behavior, sleep, and circadian system. Such modifications will ultimately lead to health-harming conditions.

## 6. Shiftwork-Related Alterations in Nervous System to Develop Psychiatric Disorders

Adversely affected nervous system by working in continuously rotating shifts may accelerate the rate of psychiatric disorder occurrence (Figure 1). Being major causes of disabilities [83], psychiatric disorders including bipolar disorder (BD), schizophrenia (SZ), and major depression disorder (MDD) impose enormous medical burdens [84–86]. The genetic alteration and uncontrolled expression of genes primarily causing the aforementioned disorders [83, 87–89] are associated with irregular continuous changing of shifts during work. Night shift work contributed toward several psychiatric disorders through circadian misalignment, sleep deprivation, and light-induced melatonin suppression [73]. Disrupted-in-schizophrenia-1 (DISC1) is an important genetic factor in serious mental disorders including SZ, BD, and MDD [90]. We will further discuss the previously mentioned psychiatric disorders one by one to provide detailed information.

### 6.1. Bipolar Disorder

BD is considered one of the severely disabling disorders. Distinctive distortions of emotion regulation make BD a severe psychiatric condition. It mainly affects emotional and social behavior with light effect on perception and thought. Being a multifactorial disorder, its risk is influenced by genetic and environmental factors [91]. Genetic and pathophysiological factors involved in the development of BD are largely unknown [92]. BD increases the risk of schizophrenia and major depression disorder which is an indication for shared genetic basis between these disorders. Although heritability has been proven, more studies are needed to investigate the genetic mechanisms [93, 94]. In BD, important genetic variants that could be affected by shiftwork either directly or indirectly are Del, ANK3 rs1938526, COMT Val158Met, DAOA, BRD1/ZBED4, BDNF Val66Met, BRD1, ASMT, CAMTA1, CCDC132, CHES1, DGKH, DRD4, HTR1A C1019G, SLC6A4, 5-HTTLPR PARD3B, PDLIM5, STin2 VNTR, KLHL3, LYPD5, MAOA T941G, MTHFR A1298C and C677T, TPH1 STAB1, HTR3B, and WNK2 [95]. They are expressed in brain and associated with CREB (cyclic adenosine monophosphate response element-binding protein). *KCNH7* R394H (rs78247304) mutation is linked to BD. *ANK3*, *CACNA1C*, an intron variant of *CACNA1C* (rs79398153), and a missense mutation of *ANK3* (N2643S) were confirmed being involved in BD [95, 96]. All these mutations are possibly enhanced in people working in several shifts or exposing to irregular light (jet lag).

### 6.2. Major Depression Disorder

MDD, a leading cause of loss in work productivity, is considered a fatal disorder. It is considered one of the most prevalent disorders that affect females more than males [97, 98]. Genetic and environmental risk factors mainly contribute to cause MDD. It is a neuroprogressive disorder in which each persisting episode increases impairment in function and sensitivity for upcoming episodes. A decrease in the GR messenger ribonucleic acid levels in hippocampus, frontal cortex, and amygdala has been observed in the patients suffering from MDD. Susceptibility to further episodes is increased by repeated illness which causes the permanent alteration in the normal functions of neurons [99] and genes [100] including MTHFR C677T and 5-HTTLPR [101]. Some of the important genetic variants associated with MDD that could be affected by shiftwork are APOE, SLC6A4, ACE, GNB3, HS6ST3, HTR1A, LHFPL2, PDE11A, DISC1, MAOA, SLC6A3 (DAT1), SLC25A21, VGLL4 BDNF, P2RX7, TPH2, PDE9A, and GRIK3 [95, 100]. Neutral amino acid transporter (SLC6A15) is a susceptibility gene for MDD [102].

## 7. Shiftwork Affects Expressions of Oncogenes to Develop Cancer

Malignancies are majorly developed by mitigation in pineal hormone melatonin by bright light at night [1]. Reduced production of melatonin, phase shift, and sleep disruption, caused by exposure to light at night, might be the possible mechanisms that cause cancer and related disorders [103]. Shiftwork causes cancer (Figure 1) via altering the clock-controlled gene expression that regulate tumor suppression [1, 104]. Per1 negatively affects the growth of tumor cell and per2 functions as tumor repressor [105]. Long-term shiftwork negatively affects IFN*γ* (interferon gamma) [106] The association of shiftwork-affected immune system with sleep deprivation increases inflammatory markers thereby causing malignancies and metabolic and cardiovascular disorders [1]. Circadian rhythm disruption by shiftwork or bright light exposure at night increases the rate of cancer and decreases the nocturnal rise in melatonin [19].

### 7.1. Breast Cancer

**Figure 2:**
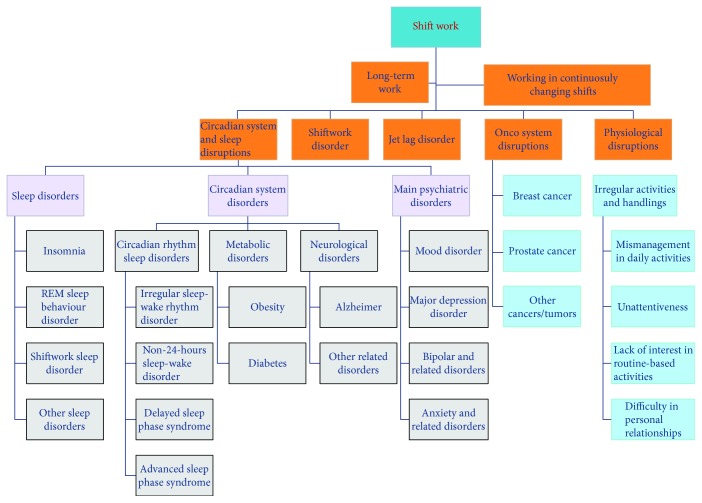
All disorders that could possibly be caused by shiftwork.

The disrupted circadian rhythm or circadian clock through shiftwork effects the development of breast cancer (Figure 2) [107]. Cell proliferation and apoptosis is regulated by approximately 7% of clock-controlled genes, including myelocytomatosis viral oncogene human recombinant (C-Myc), murine double minute oncogene (Mdm-2), and growth arrest and DNA damage-inducible alpha protein (Gadd 45a) and those encoding p53, caspases, and cyclins. Per1 is reduced in cancer tissue and its inhibition blunts apoptosis, whereas Per2 represses tumor in breast cancer and induces estradiol (E2) in mammary cells. Coexpression of Per2 with cytochrome inhibits growth of breast cancer cells. Deficiency of Per2 causes deregulation of Myc, cyclin A, cyclin D1, Mdm-2, and Gadd45a, while its dysfunctionality impairs apoptosis mediated by p53 by activating c-Myc signaling pathway. Overexpressing and downregulating Per1 or Per2 inhibits and accelerates growth of cancer cells, respectively [1]. Telomere shortening is correlated with consecutive night shift work for a long time where it increases breast cancer risk [108].

### 7.2. Colonic Cancer

Per1- and Per2-mediated tumor-repressing effect is more specific to early light and early dark phase. Per2 mutations and deregulation favor development and increases cell proliferation, respectively [1]. Altered light schedules develop mutations in PER1, PER2, and PER3 which in turn promote colonic adenoma and colonic cancer [19, 109].

### 7.3. Prostate Cancer

Shiftwork-mediated sleep disruption is associated with elevated prostate-specific antigen (PSA), indicating an increased risk of developing prostate cancer [110]. Disrupted circadian rhythms through jet lag inhibits p53, enhances Myc expression, and induces tumorigenesis in prostate tissues targeted by endocrine [1, 111, 112].

### 7.4. Ovarian Cancer

Functional analysis suggested that variation, in circadian genes including *BMAL1*, *CRY2*, *CSNK1E*, *NPAS2*, *PER3*, *REV1*, and downstream transcription factors *KLF10* and *SENP3* through disruption of hormonal pathways or changes in light/dark schedules, is associated with ovarian cancer. Silencing the expression of *BMAL1* activates p53 to prevent cell cycle arrest which indicates that *BMAL1* gene may regulate the p53 tumor suppressor pathway. Per2 inhibition, reduces estrogen receptor *α* (ER*α*) response to E2 by overexpression or enhances E2 activation [112, 113]. Its lower expression along with lower expression of *BMAL1* and *CRY1* promotes lower survival of cells in ovarian cancer [114].

### 7.5. Lung Cancer

Not only systemic but also somatic disruption of circadian rhythms mediated by jet lag alone affects c-Myc and enhances cell proliferation. Per2 and BMAL1 lose the ability of inhibiting tumor progression and hence promotes lung cancer [115].

### 7.6. Shiftwork Tolerance

The effects of shiftwork regarding tolerance and responsiveness are concerned with certain factors. The youngest and oldest individuals are affected more than individuals with middle age [116]. Females due to low tolerance develop more problems, like sleep disruptions, higher risk of mortality, disability, fatigue, and obesity, while males show more cognitive disturbances [117, 118]. In case of circadian rhythms, a low score on morningness [119] and languidness and a high score on flexibility [120] are associated positively with shiftwork tolerance.

## 8. Conclusion and Prospects

Shiftwork has gained central importance due to its detrimental effects on health. A large population of the world, including regular travelers, night shift workers, continuously faces jet lag conditions. Working in frequently rotating shifts causes several medical issues to arise, including cancer, psychiatric disorders, quick return accidents, and metabolic disorders. These conditions lasting longer may bring irreversible changes in the body, leaving no choice for affective recovery. It is impossible to avoid working in rotating shifts or prevent oneself from light exposure.

The health problems related to shiftwork are developed by disruptions in genetic expressions. To prevent or mitigate the adverse effects of shiftwork-related disorders, unveiling of genetic mechanisms and determination of related pathways are needed.

These relations and interactions could be studied in a better way through jet lag, circadian rhythms, and sleep behaviors.

The effects of shiftwork may involve a series of genes and factors majorly involved in circadian rhythm, sleep homeostasis, and the specific disorder prevention. It is considered that circadian genes have noticeable impacts on cancer controlling genes, psychiatric disorder causing genes, and metabolic disorder-related genes. But it is still needed to be investigated whether shiftwork directly affects the genes related to certain disorders or it elongates its impact via targeting other genes such as clock genes. The proper targeted medications are possible to be developed only if the mechanisms and factors causing and promoting the shiftwork effects to cause disorders have been identified.
